# Effect of Molar Mass on Critical Specific Work of Flow for Shear-Induced Crystal Nucleation in Poly (l-Lactic Acid)

**DOI:** 10.3390/polym13081266

**Published:** 2021-04-13

**Authors:** Mengxue Du, Katalee Jariyavidyanont, Ines Kühnert, Regine Boldt, René Androsch

**Affiliations:** 1 Interdisciplinary Center for Transfer-oriented Research in Natural Sciences, Martin Luther University Halle-Wittenberg, 06099 Halle/Saale, Germany; mengxue.du@iw.uni-halle.de; 2 Leibniz-Institut für Polymerforschung Dresden e.V., Hohe Str. 6, 01069 Dresden, Germany; kuehnert@ipfdd.de (I.K.); boldt@ipfdd.de (R.B.)

**Keywords:** shear-induced crystal nucleation, specific work of flow, nucleation, crystallization, poly (l-lactic acid), molar mass

## Abstract

The concept of specific work of flow has been applied for the analysis of critical shearing conditions for the formation of crystal nuclei in poly (l-lactic acid) (PLLA). Systematic variation in both time and rate of shearing the melt in a parallel-plate rheometer revealed that these parameters are interconvertible regarding the shear-induced formation of crystal nuclei; that is, low shear rate can be compensated for by increasing the shear time and vice versa. This result supports the view that critical shearing conditions can be expressed by a single quantity, providing additional options for tailoring polymer processing routes when enhanced nuclei formation is desired/unwanted. Analysis of PLLA of different mass-average molar masses of 70, 90, 120, and 576 kDa confirmed improved shear-induced crystal nucleation for materials of higher molar mass, with critical specific works of flow, above which shear-induced nuclei formation occurs, of 550, 60, 25, and 5 kPa, respectively.

## 1. Introduction

In typical polymer melt-processing routes such as injection molding, film blowing, or extrusion, the melt is subjected to shear during shaping, before its solidification by vitrification and/or crystallization [1,2,3]. During shearing, molecular segments may align and orient parallel to the direction of flow and/or be stretched, depending on the specific shear conditions applied. As a result of shearing the melt, and of the alignment and stretching of macromolecules, crystal nucleation may be enhanced in crystallizable polymers, thus accelerating the overall crystallization process, as well as affecting the supermolecular semicrystalline structure by the increased nuclei number [4,5,6,7,8,9,10,11]. As such, evaluation of the effect of shear on the crystallization process in polymer processing is an indispensable task for understanding the relationship between processing, structure, and properties [12,13,14,15,16].

Variables affecting shear-flow induced crystal nucleation in polymer melts include, besides the temperature of shear (*T*_s_) and, therefore, the viscosity (*η*) of the melt, the time (*t*_s_) and rate of shearing ($\dot{\gamma }$) [17,18,19,20,21]. Regarding the rheological parameters of the polymer, the Rouse (*τ*_R_) and reptation relaxation times (*τ*_r_) (*τ*_r_ > *τ*_R_) for describing diffusion at length-scales roughly shorter and longer than the entanglement molar mass [19,22], respectively, are of particular importance since they control the orientation (*τ*_r_) and stretch (*τ*_R_) of molecules. It is suggested that for the orientation of molecules, the melt needs to be sheared at rates $\dot{\gamma }$ > 1/τ_r_, while additional stretching only occurs for even higher shear rates of $\dot{\gamma }\;$> 1/*τ*_R_. The latter is required for observation of “oriented nuclei” and shish-kebab-type structures; otherwise, at lower rates of 1/*τ*_R_ > $\dot{\gamma }\;$> 1/*τ*_r_, there may be an expected increase in the number of point-like nuclei [21]. Regarding the effect of the shearing time, it is observed that the nuclei number increases linearly with the shearing time; however, the increase levels off when the maximum number of point-like nuclei is reached. A further increase in the shearing time may then cause fibrillation and the formation of oriented nuclei, as long as the shear rate is higher than the above-described critical value [21,23].

Shearing conditions and their effect on nucleation and crystallization can furthermore be expressed using the concept of the specific mechanical work of flow (*w*) introduced by Janeschitz-Kriegl [24], according to Equation (1):

(1) $$w=\bar{\eta }\int_{0}^{t_{s}}{\dot{\gamma }}^{2}\;dt$$

In Equation (1), $\bar{\eta }$ is the viscosity averaged over the shear rates experienced. In the reported initial study [24], it was found for isotactic polypropylene (iPP) that there is a linear relation between the nuclei number and specific work of flow when plotting in double log scale. Moreover, it was suggested that “long-lasting” deformations under low stresses can yield the same precursors as short-term deformations under high loads. Apparently, the only condition is that the applied specific work be the same [25].

The specific work of flow as a quantity to analyze shear-induced crystal nucleation in polymers has been used for the characterization of several materials, including iPP [24,26,27], hydrogenated polybutadiene [21], polyamide 66 (PA 66) [28], polyamide 11 (PA 11) [29], poly (ether ether ketone) (PEEK) [30,31], or poly (l-lactic acid) (PLLA) [32]. For iPP, besides the above-described relation between the number of point-like nuclei and the specific work of flow, a critical work of flow of 7 MPa was identified above which a transition occurs from a spherulitic morphology to a rice-grain-type morphology, consisting of randomly oriented, slightly anisotropic domains with a size of up to 3 µm [27]. For iPP, shear rate and time both contribute to the formation of nuclei, though with different degrees when shearing at low or high rates [19,23]. For PA 66 and PA 11, critical specific works of flow ($w_{c}$), defined as the work-value above which crystallization accelerates, are 40 kPa at 270 °C and 1 MPa at 190 °C [28,29], respectively, with both analyses performed at temperatures slightly lower than the equilibrium melting temperature $T_{m,0}$, thus being comparable. The reason for the largely different critical specific works of flow is yet unknown.

For PEEK, a critical specific work of flow for accelerated nucleation of 1 MPa is reported, with the increasing nuclei number saturating at around 50 MPa when shearing the melt at 328 °C—that is, at around 50 K below $T_{m,0}$ [30]. When the temperature for shearing the melt increases to 350 °C, the value at which the nuclei number saturates increases to around 135 MPa [31]. A similar saturation work of flow of around 100 MPa has been observed for PA 66 [28], and a value of 16 MPa was found for iPP [26,27].

The value of critical specific work of flow to drive the formation of (point-like) crystal nuclei in PLLA with a mass-average molar mass of 107 kg/mol is around 20 to 50 kPa at shearing temperatures of 135 and 140 °C [32]. The initial study of the effect of the specific work of flow on crystal nucleation in PLLA, however, was performed only on a single PLLA grade with a specific molar mass, and, so far, no information about the influence of the molar mass is known. In order to fill this gap, in the present study, different PLLA grades of identical and low d-isomer content, but different molar mass between 70 and 576 kg/mol, are analyzed.

While we are not aware of studies of the molar-mass effect on shear-induced nucleation of the PLLA homopolymer, investigation of the behavior of an l,d-lactic acid random copolymer (PDLLA) containing approximately 2% d-isomer co-units revealed the expected enhanced nucleation rate for higher molar-mass samples due to the increased relaxation time of the longer molecules [33] reported for many different polymers, shedding light on the molar-mass dependence of relaxation times [34,35,36,37]. Since the presence of d-isomer co-units in PLA significantly slows down the crystallization process due to their required segregation during crystal growth [38,39,40,41], however, quantitative conclusions about shear-induced crystallization of the PLLA homopolymer cannot be drawn.

In summary, the goal of the present study is to provide quantitative information about the effect of molar mass on critical conditions of shearing the melt for observation of shear-induced crystal nuclei in units of specific work of flow for PLLA not containing d-isomer co-units in the chain. As an extension of prior work on PLLA, not only the shear rate but also the shear time was used as an independent parameter to achieve flow of macromolecules, allowing us to prove/disprove the interconvertibility of these shearing parameters to obtain a specific acceleration of the crystallization rate/increase in the nuclei number. In a wider sense, this research about shear-induced nucleation of PLLA may provide knowledge about possible pathways of increasing the crystallization rate of the otherwise rather slow-crystallizing polymer [38,39,40,41] and, with that, an option to introduce new applications when the presence of crystals is desired.

## 2. Materials and Methods

*Material.* Poly (l-lactic acid) (PLLA) homopolymer grades were obtained from Corbion (Amsterdam, Netherlands) and Polysciences Europe GmbH (Hirschberg an der Bergstraße, Germany). The mass-average molar masses of the Corbion grades are 70, 90, and 120 kg/mol, determined as absolute values by gel permeation chromatography (GPC), using hexafluoroisopropanol as solvent [42]. The corresponding melt-flow rates are 50, 24, and 8 g/(10 min) (210 °C/2.16 kg), respectively [43]. The minimum l-isomer content is >99 % [43]. The mass-average molar mass and polydispersity of the Polysciences grade (Catalog number 18582) are 576 kg/mol and 1.5, respectively, measured as polystyrene equivalent by GPC, with tetrahydrofuran as solvent [44]. Before experimentation, the as-received pellets were dried in a vacuum oven at 80 °C for at least 12 h.

*Rheology.* Analysis of the effect of shearing the melt on crystallization of PLLA was done using an ARES 2000 Rheometer (TA Instruments, Newcastle, DE, USA) equipped with an environmental test chamber. The sample environment was purged with dry air, and measurements were performed in parallel-plate geometry. The diameters of the upper and lower plates were 8 and 25 mm, respectively. The surface of the lower plate was covered with Kapton^®^ film (MakerBot, Brooklyn, NY, USA) for easy removal of the sample after analysis, and the gap distance *d* between the upper and lower plates was set to 250 µm. Before setting the gap, the pellets were placed onto the Kapton^®^ film after preheating the plates to 220 °C. After the pellets melted, they were squeezed to form a disc, and any material outside the gap was removed using a spatula. Note that, in parallel-plate geometry, for a given gap distance and rotation at a constant angular velocity *ω*, the shear rate in the sample increases linearly with the radius *r* [$\dot{\gamma }=\omega \times r/d$].

**Figure 1:**
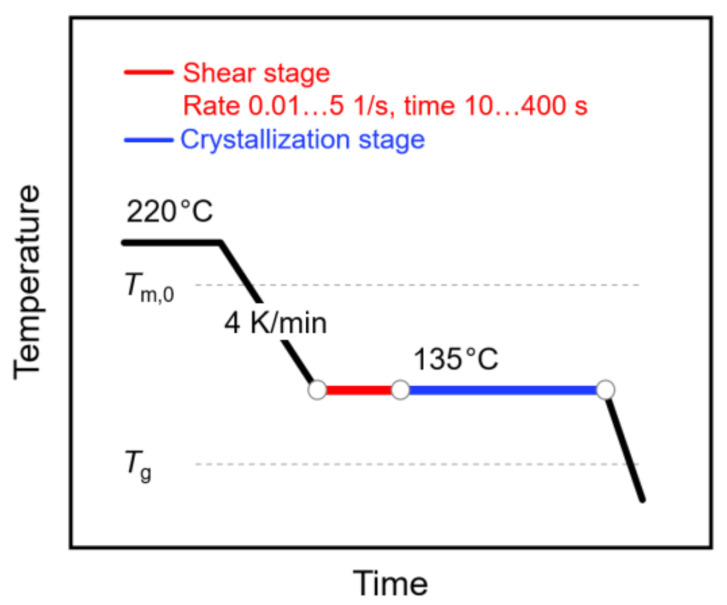
Temperature–time profile for analysis of shear-induced crystallization of poly (L-lactic acid) (PLLA). The relative positions of the equilibrium melting temperature ($T_{m,0}$) and of the glass transition temperature ($T_{g}$) are indicated with the gray dash lines.

The shear and temperature protocol of the performed experiments is shown in Figure 1. Briefly, the sample was kept at 220 °C for 5 min to erase the thermal history and then cooled to 135 °C at a rate of 4 K/min. Afterwards, the sample was sheared at rates between 0.01 and 5 1/s for 10 to 400 s (see red segment). Following the shearing step, the sample was crystallized at the same temperature for 1 h (see blue segment), before cooling to room temperature. Note that the selected crystallization time ensured completion of the crystallization process. During the crystallization stage, the sample was additionally subjected to oscillation at a frequency of 3 rad/s and a deformation amplitude of 0.05%, in order to measure the viscosity needed for calculating the specific work of flow (see Equation (1)) and for following the increase in the crystallinity. Note that analysis of the molar mass of a selected sample, before and after the thermo-mechanical treatment, proved the absence of degradation.

*Polarized-light optical microscopy (POM)*. POM was employed for analysis of the semicrystalline morphology of the discs prepared in the rheometer at the micrometer-length scale. We used a DMRX microscope (Leica, Wetzlar, Germany), operated in transmission mode, with thin sections placed between crossed polarizers. Thin sections with a thickness of 10 µm were prepared using a rotary microtome (Slee, Mainz, Germany) equipped with a tungsten carbide knife. Sections were taken from the edge of the discs, with the cutting direction parallel to the flow direction.

*Wide-angle X-ray scattering (WAXS).* WAXS was used to investigate the crystal structure and orientation as a function of the radial position in the disks from the edge to the center. The analysis was performed at room temperature in transmission mode, employing a D8 Discover X-ray diffractometer (Bruker, Billerica, MA, USA), equipped with a Vantec 500 area detector, and using Cu K_α_ radiation. The diameter of the circular beam was 0.5 mm and the exposure time was 30 min. The sample-to-detector distance was 149.7 mm.

## 3. Results and Discussion

### 3.1. Reptation Relaxation Time

**Figure 2:**
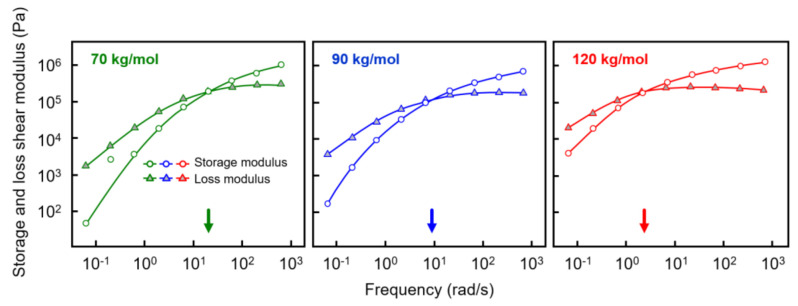
Storage (open circles) and loss shear modulus (gray triangles) of PLLA of molar mass of 70 (**left**), 90 (**center**), and 120 kg/mol (**right**), as a function of frequency at 135 °C. The colored arrows at the frequency axes indicate the relaxation times of the various PLLAs.

Prior to shear-induced crystallization experiments, the rheological properties of the various samples were investigated in order to obtain information about the reptation relaxation time at the temperature of interest of 135 °C. Figure 2 shows the storage shear modulus *G*’ (white circles) and loss shear modulus *G*’’ (gray triangles), both plotted as a function of the frequency of deformation with an amplitude of 0.05%. Most important in the context of estimating relaxation times is the observation of a distinct shift in the crossover frequency, defined as the frequency where *G*’ and *G*’’ are equal, to lower values if the molar mass increases (see also the arrows at the frequency axes). For the analyzed polymers with molar masses of 70, 90, and 120 kg/mol, reptation times of 0.05, 0.11, and 0.5 s were obtained as the inverse of the crossover frequency, respectively [45,46,47]. As such, tube relaxation times increased with molar mass, or, in other words, the time for the molecular segments to return into their equilibrium shape after perturbation increased, thus requiring a lower rate of deformation for orientation. Again, while the relation between molar mass and shear-induced nuclei formation has been confirmed in numerous studies on different polymers [34,35,36,37], dedicated work focusing on the PLLA homopolymer is lacking.

### 3.2. Crystallization Kinetics and Semicrystalline Morphology of PLLA of Different Molar Mass after Shearing at Different Rates for 10 s

**Figure 3:**
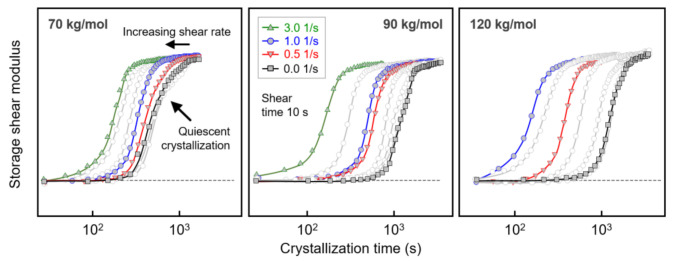
Storage shear modulus of PLLA with molar masses of 70 (**left**), 90 (**center**), and 120 kg/mol (**right**) as a function of crystallization time, after shearing the melt at different rates at 135 °C for 10 s. Note that for PLLA with a molar mass of 120 kg/mol, shearing the melt at 3 1/s led to crystallization during the 10-s-shear step, with the data therefore omitted.

The left, center, and right sets of curves in Figure 3 show the evolution of the storage shear modulus *G*’ during crystallization of PLLA with molar masses of 70, 90, and 120 kg/mol, respectively. The data were collected after shearing the melt at 135 °C for 10 s at different rates between 0 (black/gray squares) and 3 1/s (green triangles), as indicated in the legend. Above the glass transition temperature, the storage modulus increased during crystallization with the crystallinity, [48] and the generally observed shift of the curves to shorter crystallization times with increasing shear rate reflecting the increased number of crystal nuclei. Regarding the effect of shear on crystal nucleation, most important was the detection of molar-mass-dependent critical shear rates above which shear-induced crystal nucleation occurred and crystallization proceeded faster, as quantified below. Furthermore, within the analyzed range of shear rates, the curve shape/slope, qualitatively, was preserved, indicating a similar nucleation mechanism and spherulitic crystal growth [23].

**Figure 4:**
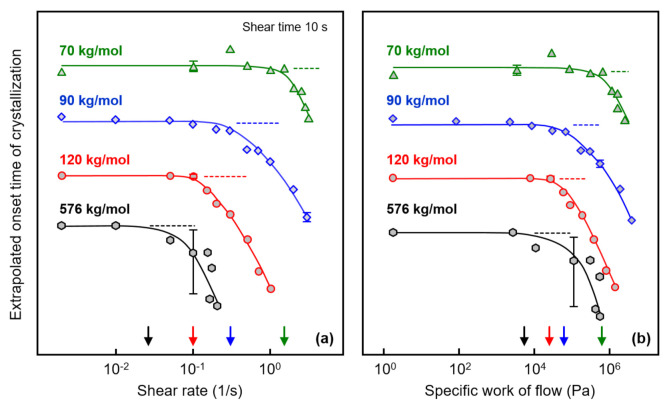
Extrapolated onset time of crystallization of PLLA of different molar mass as a function of the shear rate (**a**) (left) and as a function of the specific work of flow (**b**) (right). The dashed lines indicate zero-shear onset times, and the arrows at the shear-rate/specific-work-of-flow axes indicate critical conditions above which shear-induced crystal nucleation occurs. Error bars are shown for selected data only, being typically lower than the symbol size for the Corbion-grade samples (70, 90, and 120 kg/mol).

Figure 4a shows the extrapolated onset times of crystallization, defined as the intersection of the highest slope tangent of the modulus curve and the baseline (see gray dash lines in Figure 3) as a function of the shear rate, to obtain quantitative information about the correlation between the shear rate and the crystallization rate. In this plot, data observed on an additional sample with a molar mass of 576 kg/mol are included, obtained from a different source than the other three samples. Note that the various curves are shifted relative to each other along the crystallization–time axis for improved illustration of the effect of shear on crystallization kinetics. The plateau values at low/zero shear rate represent extrapolated onset times of crystallization of the quiescent melt, being around 300, 700, 800, and 880 s for the analyzed polymers with molar masses of 70, 90, 120, and 576 kg/mol, respectively. Critical shear rates above which crystal nuclei form by shear were estimated for the various PLLA homopolymers of different molar mass as the shear rate value at which the crystallization onset time deviates from the value observed for the quiescent melt, with the latter indicated with the horizontal dash lines. Accordingly, the critical shear rates for PLLA with molar masses of 70, 90, 120, and 576 kg/mol are around 1.5, 0.2, 0.1, and 0.02 1/s, respectively, indicated by the arrows at the shear rate axis.

The extrapolated onset time of crystallization is re-plotted as a function of the specific work of flow in Figure 4b, with the latter calculated using Equation (1). As such, the critical specific work of flow above which shear-induced nucleation and faster crystallization occur decreased systematically with increasing molar mass from 550 kPa for the sample with a molar mass of 70 kg/mol to 5 kPa for the sample with a molar mass of 576 kg/mol (see also the arrows at the specific-work-of-flow axis).

**Figure 5:**
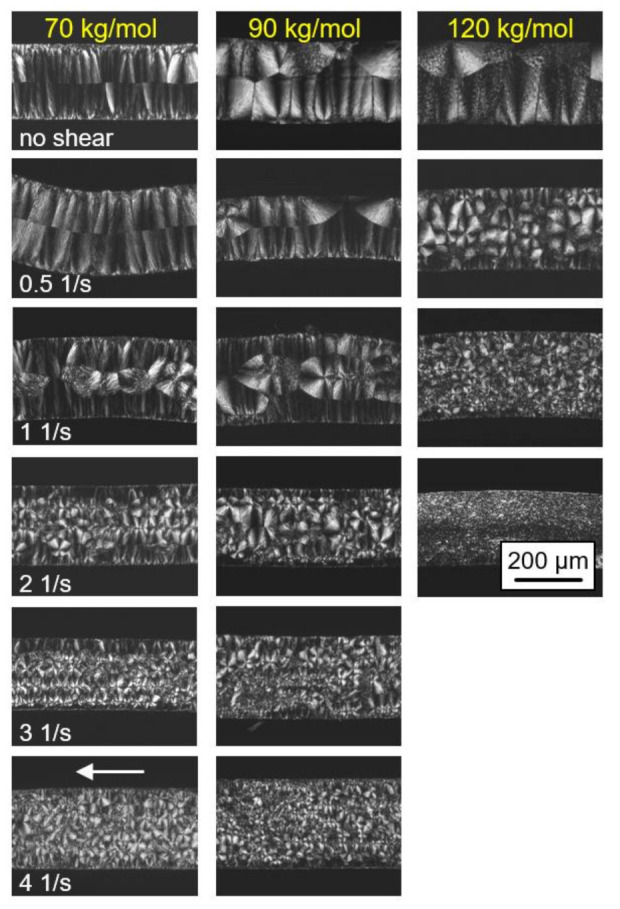
Polarized-light optical microscopy (POM) micrographs of PLLA of different molar masses (see left to right column), taken after shearing the melt at 135 °C for 10 s at different rates from 0 (top row) to 4 1/s (bottom row) and isothermal crystallization at identical temperatures until completion. Images were taken at room temperature, and the flow direction is indicated with the white arrow in the bottom left image. Note that images for the sample with a molar mass of 576 kg/mol are not shown due to difficulties in removing the films from the rheometer.

The effect of shear on the final semicrystalline morphology of the PLLA samples of different molar mass, as observed by POM, is demonstrated in Figure 5. The left, center, and right columns show images obtained on PLLA with molar masses of 70, 90, and 120 kg/mol, respectively, while the various rows are associated with different shear rates, from zero shear (top row) to 4 1/s (bottom row). Again, the images were collected from thin sections taken parallel to the flow direction at the perimeter of the sheared discs, with the flow direction indicated by the white arrow in the bottom left image.

In the rheometer, the top of the samples was in contact with the metal plate while the bottom faces were in contact with the Kapton^®^ film. However, the images reveal no effect of the different contact materials, as the structure appears similar at the top and bottom surfaces. Crystallization at quiescent conditions (see top row images) began at the top and bottom surfaces of the discs, as is concluded from the observed symmetry of the morphology and the directed spherulitic growth from surface nuclei towards the center layer. With increasing molar mass, the number of surface nuclei and, consequently, of spherulites, decreased, being in agreement with the slower crystallization detected with the data of Figure 3. With increasing shear rate, additional nuclei appeared in the center of the discs, with the effect of molar mass in particular obvious in the samples sheared at 0.5 1/s. Under this condition, additional nuclei were only observed for the higher-molar-mass PLLA sample. With a further increase in the shear rate, the nuclei number gradually increased, with the trend that the nucleation effect remained larger in the higher-molar-mass sample. It is worth noting that within the investigated range of shear rates, mainly an increase in the number of arbitrarily positioned, point-like nuclei was observed, and only occasionally were aligned/row nuclei detected.

**Figure 6:**
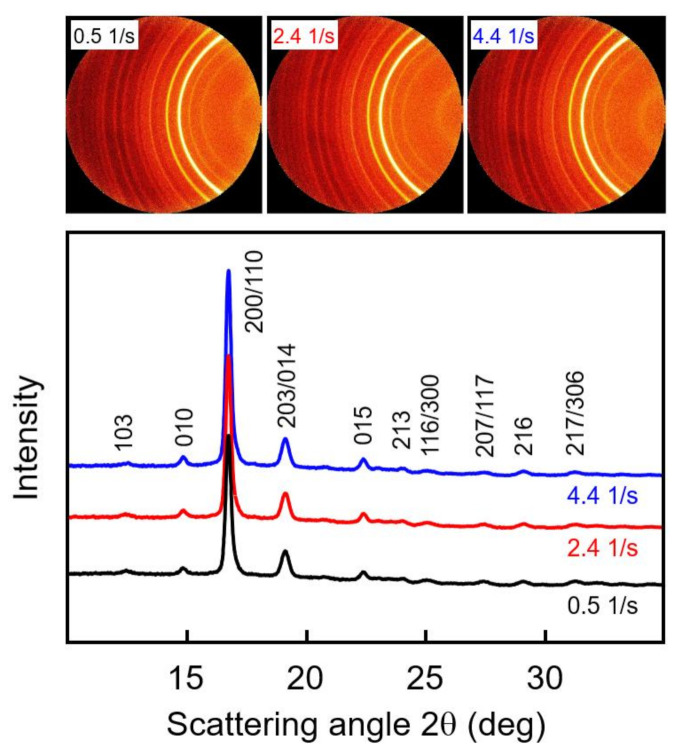
Two-dimensional wide-angle X-ray scattering (WAXS) patterns of PLLA with a molar mass of 70 kg/mol, acquired at different radius positions of 0.4, 1.9, and 3.5 mm of a crystallized disc sheared at 135 °C for 10 s with a rate of 5 1/s (**top**), and azimuthally averaged intensity as a function of the scattering angle 2θ (**bottom**). Diffraction peaks were indexed based on information available in the literature [49,50].

WAXS measurements of samples subjected to shear before crystallization were performed to obtain information about possible crystal orientation and the prevailing crystal polymorph. The top part of Figure 6 shows examples of selected two-dimensional WAXS patterns of PLLA with a molar mass of 70 kg/mol, acquired at different radius positions of 0.4, 1.9, and 3.5 mm of a crystallized disc sheared at 135 °C for 10 s with a rate of 5 1/s, and the bottom graph is a plot of the azimuthally averaged intensity as a function of the scattering angle 2θ. The shear rate is a linear function of the disc radius, and, as such, the WAXS frames are associated with shear rates of 0.5, 2.4, and 4.4 1/s. Note that shearing the melt at 0.5 1/s did not cause shear-induced nucleation, thus representing quiescent-melt crystallization. The frames show sets of Debye-Scherrer rings with a uniform intensity distribution, indicating a random orientation of crystals in the analyzed plane perpendicular to the disc surface. This result agrees with the observation of an increased number of point-like nuclei due to shear and mainly spherulitically grown crystals, even at the outer perimeter of the discs (see bottom left image in Figure 5), and an absence of large amounts of row nuclei or shish-like structures. The radial intensity curves, obtained by azimuthal averaging of the two-dimensional WAXS patterns, shown in the bottom graph, furthermore revealed, by the angular position of the scattering peaks, the exclusive presence of α-crystals. The observed scattering peaks are indexed in Figure 6, based on α-crystal structure information available in the literature [49,50].

### 3.3. Interconvertibility of Shear Rate and Shear Time for Shear-Induced Nuclei Formation

**Figure 7:**
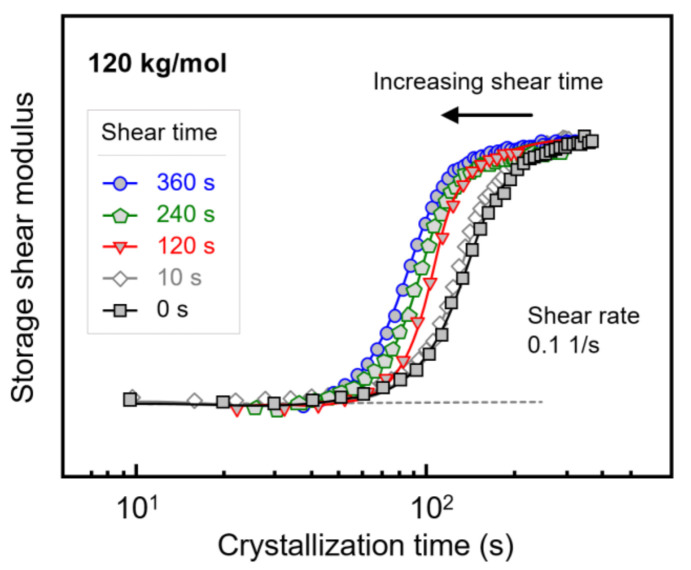
Storage shear modulus of PLLA with a molar mass of 120 kg/mol as a function of the crystallization time, after shearing the melt at 0.1 1/s at 135 °C for different shear times.

Figure 7 shows the evolution of the storage shear modulus during isothermal crystallization of PLLA with a molar mass of 120 kg/mol, after shearing the melt at a rate of 0.1 1/s for different times between 0 and 360 s. Note that the shear time is included in the crystallization time. Despite the rather low shear rate, shearing for 10 s hardly affected the crystallization rate (see Figure 3 and Figure 4), there is observed a shift in the storage modulus curves to shorter crystallization times with increasing shear time, indicating an increase in the number of point-like nuclei [23,51]. Similar experiments were performed on PLLAs of lower molar mass, yielding qualitatively similar observations. Again, the purpose of variation in the shear time served to evaluate whether shear rate and shear time are interconvertible regarding their effects on crystallization kinetics within the concept of the specific work of flow. For this reason, Figure 4b, showing the crystallization onset time as a function of the specific work of flow for samples subjected to different shear rate only, was expanded to include data observed after shearing for different times.

**Figure 8:**
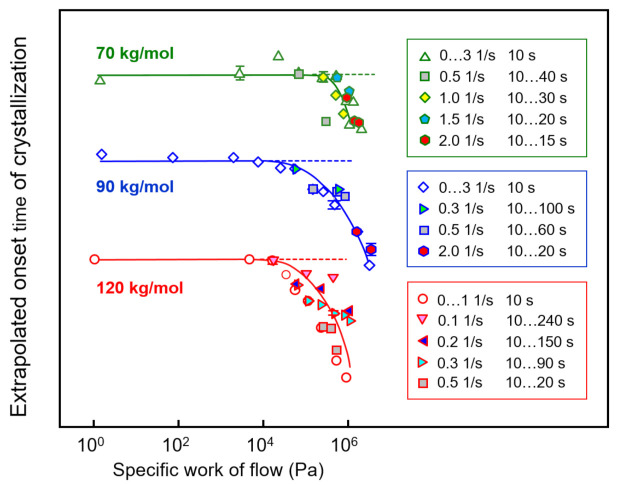
Extrapolated onset times of crystallization of PLLA of different molar mass of 70, 90, and 120 kg/mol (from top to bottom) as a function of the specific work of flow. The dashed lines indicate the zero-shear crystallization onset time. Error bars are shown for selected data only, being typically lower than the symbol size. Data from the sample with a molar mass of 576 kg/mol are not shown due to low reproducibility.

Extrapolated onset times of crystallization of PLLA of different molar mass of 70, 90, and 120 kg/mol are shown as a function of the specific work of flow in Figure 8, with the curves vertically shifted relative to each other for improved visibility. The different data sets are based on shear experiments employing different combinations of shear time and rate to obtain a single specific-work-of-flow value (see legends), in order to analyze their interconvertibility for the acceleration of crystal nucleation. The data clearly revealed that within the analyzed range of shear parameters, indeed, it was possible to achieve an interconversion of rate and time to achieve a similar accelerating effect on crystallization.

## 4. Conclusions

In the present study, the effect of molar mass of PLLA homopolymers (d-isomer content of approximately 0%) on shear-induced nucleation was analyzed. At given temperatures and times of shear, it was found that the critical shear rate, above which additional crystal nuclei form, decreased with molar mass. Specifically, for PLLA of molar masses of 70, 90, 120, and 576 kg/mol, critical shear rates of 1.5, 0.2, 0.1, and 0.02 1/s were detected, respectively, when shearing the melt for 10 s at 135 °C. In an extension of numerous works about shear-induced crystallization of PLLAs, the concept of specific of flow was applied, yielding critical work values of 550, 60, 25, and 5 kPa, from low to high molar mass. Moreover, if the shear rate is higher than a specific value (not evaluated in the present study), then the square of the shear rate is interconvertible with the shear time (see Equation (1)). Importantly, if plotting crystallization onset times as a function of *w*/$\dot{\gamma }$, instead of as a function of *w*, then the different combinations of $\dot{\gamma }$ and *t*_s_ do not yield a single relationship. The effect of shearing the melt on the crystallization process has been confirmed by POM, which shows a higher number of point-like nuclei/smaller spherulites after shearing the melt at supercritical conditions. The critical specific work of flow of PLLA (5 to 550 °kPa, depending on the molar mass) is low, similar to the case of PA 66 (40 kPa), if compared with iPP (7 MPa), PEEK (1 MPa), or PA 11 (1 MPa). The reason is unknown, requiring further research efforts to unravel the effects of the molecular architecture and shearing conditions.

## Data Availability

Data available on request.
